# Comparison between Recurrent Networks and Temporal Convolutional Networks Approaches for Skeleton-Based Action Recognition

**DOI:** 10.3390/s21062051

**Published:** 2021-03-15

**Authors:** Mihai Nan, Mihai Trăscău, Adina Magda Florea, Cezar Cătălin Iacob

**Affiliations:** Faculty of Automatic Control and Computers, University POLITEHNICA of Bucharest, RO-060042 Bucharest, Romania; mihai.nan@upb.ro (M.N.); mihai.trascau@upb.ro (M.T.); cezar_catalin.iacob@stud.acs.upb.ro (C.C.I.)

**Keywords:** action recognition, sequence-to-sequence, temporal convolutional networks, recurrent networks

## Abstract

Action recognition plays an important role in various applications such as video monitoring, automatic video indexing, crowd analysis, human-machine interaction, smart homes and personal assistive robotics. In this paper, we propose improvements to some methods for human action recognition from videos that work with data represented in the form of skeleton poses. These methods are based on the most widely used techniques for this problem—Graph Convolutional Networks (GCNs), Temporal Convolutional Networks (TCNs) and Recurrent Neural Networks (RNNs). Initially, the paper explores and compares different ways to extract the most relevant spatial and temporal characteristics for a sequence of frames describing an action. Based on this comparative analysis, we show how a TCN type unit can be extended to work even on the characteristics extracted from the spatial domain. To validate our approach, we test it against a benchmark often used for human action recognition problems and we show that our solution obtains comparable results to the state-of-the-art, but with a significant increase in the inference speed.

## 1. Introduction

The problem of recognizing people’s actions is very complex because it depends on many factors. This subject became one of the most important research topics in the field of computer vision due its wide applicability in practical applications. Recently, more and increasingly larger datasets appeared in the field of human action recognition, which were able to facilitate the construction of better solutions for this problem. An action can be seen as a series of human body movements. Thus, there are several ways in which these movements, that define a human action, can be recorded—as a video clip (a set of RGB images), by recording a series of depth maps, or in the form of a data structure storing the positions of many joints for each time frame, either representing a time-dependant 3D mesh of the visible human body surface or even just a time-dependent graph of articulation points that describes a simplified model of a human skeleton or other combinations.

**Figure 1:**
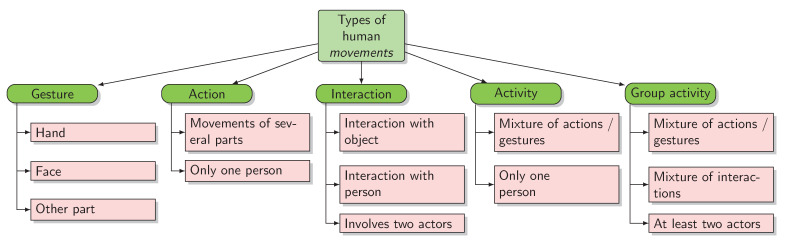
Characteristics of the main types of human movements.

An important observation that needs to be clarified, before presenting the difficulties and challenges of the problem, is related to the difference between the different types of human movements. There are no unanimously accepted definitions, but we consider that the differences between these two concepts are those presented in Figure 1, extracted from the definitions provided by Aggarwal et al. [1]. It is relevant to make this distinction because this paper addresses the problem from the perspective of classifying human actions using skeletal data. The NTU RGB+D dataset [2], containing samples describing actions and interactions, was used to train the models presented in this paper. The samples that describe interactions are viewed only from the perspective of interacting with another person because the Kinect sensor does not capture information that illustrates the interaction with other objects.

1. Gesture—this category is based on face, hand or other parts movements, representing elementary movements of a person’s body part. This category presents the smallest challenge, being about a single part of the body. Thus, there are not many types of gesture categories. Each type can be differentiated from each other depending on the part of the body that is involved.
2. Action—actions are single-person activities that may be composed of multiple gestures organized temporally. Most datasets [2,3,4,5,6] and most proposed solutions [7,8,9,10,11] are focused on this category.
3. Interaction—interactions are human activities that involve two or more persons and/or objects. For some ways of encoding data, it is difficult to represent the entire information necessary to describe this category. For example, it can be difficult to recognize interactions involving objects using skeletal data.
4. Activity—this is a complex category. An activity can be perceived as a series of several actions. Unfortunately, there are not many datasets that provide information for this category. Depending on the order of the actions in the sequence, we can have one activity or another, so the temporal component has an important role.
5. Group activity—it can be a mixture of gestures, actions, or interactions. The number of performers can be at least two or more, and they can interact with objects.

The main challenge that arises for the problem of recognising human actions is related to the development of a module capable of running in real time. Many approaches have been proposed to this problem, but most of the time, they have only been tested on certain benchmarks as it is very difficult to implement a model that can generalize and function in any conditions. For example, methods based on RGB images may become dependent on the environment in which the training set samples were collected. Skeleton-based approaches depend very much on the correctness of the skeletons.

The problem of recognising human actions depends on many factors, and each of them can be decisive in determining the result. Some of these factors are actually related to the action to be classified (e.g., duration of the action, speed with which the action is performed, height of the person performing the action, brightness, the quality of the data collected) and others occur when we want to integrate such a module in a system that runs in real time (e.g., detecting the moment when the action starts, detecting the moment when the action ends, checking whether or not an action of interest has happened, the time required to predict the action).

From the perspective of machine learning, the problem of recognizing human actions can be reduced to a sequence classification problem through which a model capable of assigning a label (an action) is developed for a sequence received as input. In other words, this problem is a many-to-one sequence modelling problem.

Our ultimate goal is to create a proactive social robot. One of the important aspects that should be achieved for this final purpose is the development of a high-performance human action’s recognition module. This module must be robust and with a short inference time. In this paper, we present an approach for such a module that deals with both spatial and temporal dependencies. We focus on recognizing human actions based on data extracted using the Kinect sensor because the processing operation for such data is computationally efficient. Also, it is relevant to notice that this representation can compress a large amount of information. In [12], we showed that such a module can be integrated into a robotic framework.

In our endeavour, we considered two types of architectures: Recurrent Neural Networks (RNNs) and Temporal Convolutional Networks (TCNs), as the most used architectures for human action recognition from skeletal data.

The approach for which we obtained the best results is the one based on TCNs. Regarding computational capabilities, TCNs, much like other convolutive networks, can take advantage of asynchronous and/or parallel computational architectures, most notably Single Instruction Multiple Data (SIMD) vectorial computing units and FPGAs. Carreras et al. [13] proposed an implementation variant optimized for Field Programmable Gate Arrays (FPGAs). This could indicate that the proposed approach could be extended to be usable on Internet of Things (IoT) devices that have limited memory and lower computing power.

The main contributions of our current work include the following:

- We propose two approaches that obtain results comparable to state of the art for the human action recognition problem, but which have a shorter inference time. These two approaches are based on a simplified machine learning architecture.
- We extend the classical version for the TCN unit by replacing 1D convolutions with 2D convolutions that use dilated factor for both temporal and spatial domains. This aspect helped us to obtain a better performance for human action recognition because the spatial dimension is important.
- We present two ways of rearranging the skeleton joints that help the model to extract more relevant spatial features.

## 2. Related Work

### 2.1. Skeleton-Based Human Action Recognition

Certain patterns that are present in the moves of a skeleton model that roughly maps the structure and motor behaviour of a real person might contain sufficient data for human action classification. This skeleton can be represented as a graph with its nodes in the shape of a human that change their spatial coordinates through time. Because of their apparent simplicity and because those structures densely pack information, unlike images, for example, many researchers tried using such graphs to solve the tricky problem of human actions recognition.

#### 2.1.1. TCNs-Based Approaches

Recently, researchers proposed approaches that replace the layers based on RNNs with layers based on Temporal Convolutional Networks (TCNs) for several types of sequence modelling problems [14,15,16,17]. The concept of TCN was first introduced by Lea et al. [10]. They developed it to analyse long-range patterns. The main properties of this type of neural network are the following:

- the output size is equal to the input size (we can keep the length of the sequence as in the case of a recurrent neural network);
- for the characteristics from step *t*, no future information is taken into account, which can be ensured by using a causal convolution;
- in order to ensure a large receptive field, dilation factor is used for causal convolutions.

Yan et al. [8] attempted a new method of extracting useful information from dynamic skeletons in the search for a better predictor regarding human actions. They used a Spatial-Temporal Graph Convolutional Networks (ST-GCN) to combine both spatial data and temporal data into one big predictive system. Firstly, they started with a spatial graph neural network that can look at the skeleton model in one single frame and then they added the temporal architecture of TCNs over this structure, to let the model deal with the full 3D+Time dimensionality of the problem. A Graph Convolutional Network (GCN) is a multi-layered convolution that acts upon key nodes in a graph and their neighbours, similarly to how a classic CNN acts. Problems and their corresponding solutions presented in this article are choosing partition strategies, using learnable edge importance weighting, choosing a dataset with good evaluation metrics and designing a trainable architecture. Starting from this type of architecture, multiple variants have been proposed [18,19,20,21]. These approaches have shown that much better results can be obtained using GCNs instead of classic CNNs when working with time sequences containing skeletal data.

Jia et al. [9] introduced a new variant of feature representation for the skeleton-based human action recognition problem. They divided the usual vector representation for a human skeleton into five relevant joint subgroups, namely the left arm, right arm, left leg, right leg and trunk and then those parts were linked together into a whole body with the head. For each group or subgroup of joints, they chose a parent joint and they described each of the other joints in relation to their parent joint, and then they took the parent joints for each subgroup and represented them in relation to a root node, which they chose as the head node in their implementation. Using both the classic representation and this novel representation, they created a two-stream TCN architecture with an intra-frame stream and an inter-frame stream. For each stream, they used 12 residual blocks containing a batch normalisation layer followed by a parametrised leaky ReLU function followed by a convolutional layer, followed by another four layers of the same type and in the same order as those first four. Regarding the full architecture, before those 12 residual blocks, they placed a convolutional layer for size conversion. After the residual blocks, they placed a global average pooling layer, followed by a fully connected softmax layer using cross-entropy and then the results are averaged.

Lea et al. [10] used an Encoder-Decoder TCN approach for human action segmentation throughout a video. They showed that TCN architectures can accomplish the same functions as Long Short-Term Memory (LSTM) and can be over an order of magnitude faster to train than LSTM. Firstly, the proposed architecture has an Encoder-Decoder TCN which is the centrepiece of this approach and uses only convolutions, pooling and upsampling to capture the long-range temporal patterns. Secondly, they used a dilated TCN with filters inspired by WaveNet [22] to further combine activations from different layers using skip and residual connections.

Aksan and Hilliges [23] proposed a new variant of TCN that has stochastic layers. This type of TCN was inspired by Stochastic RNN architectures where the hidden state updates are in accordance to a hidden Markov model. In TCNs, the hidden state is replaced by causally independent TCN layers, thus the sense of directional order has to be given by the way layers are linked into the convolution. To solve this impediment and allow for TCN to have the same modelling capabilities as Stochastic RNNs while preserving the parallelism of TCNs without needing dynamic links within the convolutive operation, the authors proposed a Stochastic TCN architecture that uses special stochastic layers with latent variables. The stochastic latent variables sit in a multi-layer hierarchy. These variables are independent between time steps but are conditioned vertically, in the same time step.

Plizzari et al. [24] proposed a novel Spatial-Temporal Transformer network (ST-TR) which models dependencies between joints using the Transformer self-attention operator. They use a two-stream architecture where a spatial self-attention module is used to model intra-frame relations and a temporal self-attention module is used to model inter-frame correlations. At the basis of this approach lays a graph convolutional network that can be applied over non-grid data structures. But GCNs cannot solve everything by themselves. To deal with the ordering and causality problems, Plizzari et al. shaped the GCN structure into a transformer topology which follows a general encoder-decoder pattern but relies solely on multi-head self-attention and it is able to replicate the behaviour of classic RNNs. This architecture topology is used by the two previously mentioned modules (the Spatial Self-Attention and the Temporal Self-Attention).

Song et al. [7] presented an approach that uses “bottleneck” blocks to build a ResGCN/ PA-ResGCN architecture. They also use multiple-input branches and a newly proposed Part-wise Attention block that they demonstrate to enhance the performance of the module and its explainability as well. A bottleneck is a nifty block structure that has 1×1 convolutional layers before and after the main convolutive layer, thus significantly reducing the number of feature channels. The Part-wise Attention (PartAtt) block is inspired by other part-based models like Split Attention (SplitAtt) used in the ResNeSt [25] model, which usually aims towards extracting features from individual body parts. The main differences between PartAtt and other similar part attention methods are that PartAtt is based upon global contextual feature maps obtained through average pooling over the entire temporal sequence, while other approaches work with each temporal frame independently and PartAtt works on whole body parts, not just joints. SplitAtt, for example, splits feature channels into cardinal groups but PartAtt associates instead the body parts with cardinal groups.

#### 2.1.2. RNNs-Based Approaches

The first approaches [26,27,28,29,30,31] based on deep learning consisted of a model composed of a feature extraction module, which contained linear or convolutional layers, a component that analysed sequential data using one or more recurrent neural networks and in the end the data was passed through the classification module. These approaches obtained satisfactory results, but in general they were architectures with a very large number of parameters, being difficult to apply in real-time running scenarios.

Li et al. [32] proposed an approach for the recognition of human actions, starting from skeletal data, which combined spatio-temporal graph convolution (ST-GCN) and graph-temporal LSTM (GT-LSTM). The first component does not contain a large number of layers to ensure a large receptive field because this component is not specialized in identifying long-term dependencies. The second component consists of two modules—Graph-LSTM and Temporal-LSTM. The first module performs a fusion between the features determined by the first component of the pipeline, and the second module is the equivalent of the classic LSTM applied on the merged data into temporal feature sequences. The proposed approach was validated on the RGB+D NTU dataset (the first 60-class variant [6]), and the best results were obtained for a temporal kernel size equal to 5.

Huang et al. [33] introduced a new recurrent network model—long-short graph memory network (LSGM). This type of neural network tries to combine the properties of GCN type layers with LSTM type layers. Thus, they obtained a neural model capable of capturing even spatial information that can be used for sequential data that can be represented as a graph. The method proposed by them also contained a module called Graph Temporal-Spatial Calibration (GTSC) consisting of a component based on a temporal attention mechanism and a spatial calibration component.

Introduction of attention mechanisms was an intuitive choice because there may be joints that are relevant for specific actions or there may be actions for which a certain sequence of frames is specific. Si et al. [34] proposed a hierarchical model based on a specific attention mechanism: Attention Enhanced Graph Convolutional LSTM Network. This mechanism is capable of capturing the relationships that exist between the spatial and temporal domains. The proposed method uses a recurrent LSTM network to map the features, determined by a linear layer for each joint, in a larger dimensional space.

The approaches proposed by us differ from the existing ones by using a simpler architectural model that can obtain satisfactory performances and a high inference speed. Modules based on TCN have been previously proposed in the literature, which specialised in the analysis of both spatial and temporal areas of interest. As previously presented, these modules are obtained by combining TCNs with GCNs [35,36,37]. In contrast, we propose an architectural model that is able to achieve similar results using only extended TCN units.

Bai et al. [38] made a comparison between the performance obtained for representing sequences based on TCN-based models and on RNN-based models. This evaluation was an empirical one and revealed that a simpler model (with a smaller number of parameters) based on convolutional layers can exceed the performance of a more complex model based on recurring networks. In this paper, we compare these two types of networks from the perspective of the human action recognition, showing that in this case the problem is one for which the spatial dimension is important.

## 3. Proposed Methods

We started from the performances obtained by the architectures we proposed in a previous paper [11] verified on a more varied dataset, namely NTU RGB+D v2 [2]—which contains 60 additional classes. Thus, we found that the features we had considered for these models (the 3D coordinates of the joint or the coordinates together with the velocity and acceleration) become insufficient when it comes to a dataset with such a wide variety. In contrast, the methods presented in this paper use the features proposed by Song et al. in [7]: joint positions (relative and absolute), bone features (lengths and angles) and motion velocities (one or two temporal steps). Starting from these features, we will present in what follows a series of architectural models capable of solving the problem of recognising human action based on skeletal data.

**Figure 2:**
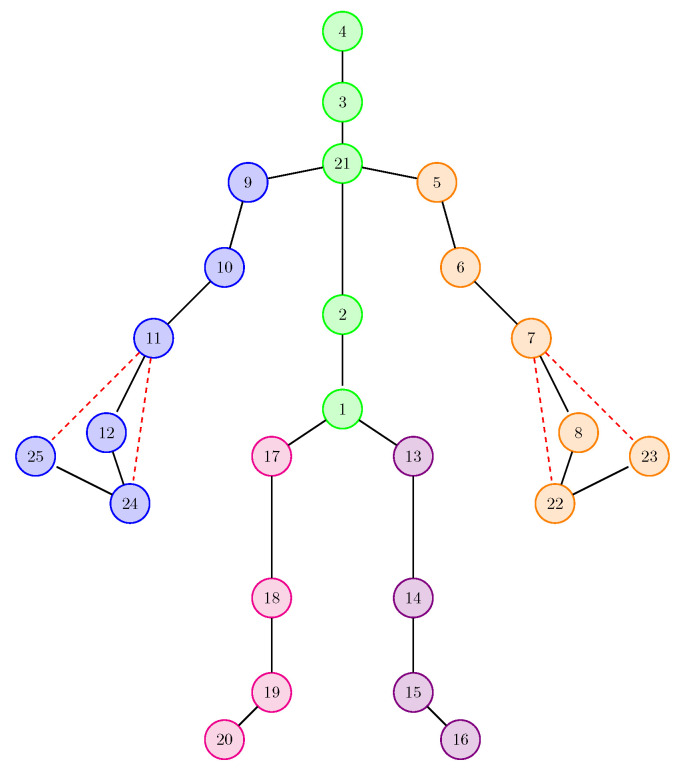
The human skeleton in the format generated by the Kinect sensor.

An action is represented in the form of a sequence of frames. For each frame, the skeletal data for one or two people are known (depending on the type of action). Each skeleton is composed of 25 articulation points (referred to as joints). Figure 2 shows how these joints make up the human body.

### 3.1. Data Processing

Processing is a very important step because extracting relevant information can help the network correctly differentiate actions. Starting from the coordinates that are provided in the dataset for each joint and applying the pre-processing methods proposed by Song et al. [7], we were able to obtain information corresponding to the three important channels: joint positions, velocities and bone features. The pre-processing step implies taking the raw skeleton data represented by all node coordinates and converting it into smoothed and normalized coordinates, approximate inter-frame velocities and approximate angles between segments at each adjacent joint.

An X vector is read from the dataset for each sample, where $X\in R^{C\times T\times V\times M}$ (C=3—number of coordinates, *T*—number of frames, V=25—number of joints, M∈{1,2}—number of people). For the joint-branch, for each joint, 3 values are added, determined based on the difference between the coordinates of the joint $j_{i}$ and those of the joint considered center of gravity $j_{c}$:

$$joint\_features_{j_{i}}=\left(x_{j_{i}},y_{j_{i}},z_{j_{i}},x_{j_{i}}-x_{j_{c}},y_{j_{i}}-y_{j_{c}},z_{j_{i}}-z_{j_{c}}\right)$$

(the center of gravity was considered the joint with the index 1 in Figure 2 – *base of the spine*). For the velocity-branch, the differences between the coordinates of the joint at frame t+2 and those at frame *t* were determined, as well as the differences between the coordinates of the joint at frame t+1 and those at frame *t*:

$$velocity\_features_{j_{i}}^{t}=\left(x_{j_{i}}^{t+2}-x_{j_{i}}^{t},y_{j_{i}}^{t+2}-y_{j_{i}}^{t},z_{j_{i}}^{t+2}-z_{j_{i}}^{t},x_{j_{i}}^{t+1}-x_{j_{i}}^{t},y_{j_{i}}^{t+1}-y_{j_{i}}^{t},z_{j_{i}}^{t+1}-z_{j_{i}}^{t}\right)$$

For the bone-branch, we also have 6 features that include the 3 lengths and the 3 values of the angles for the X,Y,Z axes:

$$bone\_features_{\left(j_{u},j_{v}\right)}=\left(x_{j_{u}}-x_{j_{v}},y_{j_{u}}-y_{j_{v}},z_{j_{u}}-z_{j_{v}},a_{(j_{u},j_{v}),x},a_{(j_{u},j_{v}),y},a_{(j_{u},j_{v}),z}\right)$$

where joints $j_{u}$ and $j_{v}$ are adjacent, $l_{(j_{u},j_{v}),x}=x_{j_{u}}-x_{j_{v}},l_{(j_{u},j_{v}),y}=y_{j_{u}}-y_{j_{v}},l_{(j_{u},j_{v}),z}=z_{j_{u}}-z_{j_{v}}$ and

$$a_{(j_{u},j_{v}),x}=\arccos\left(\frac{l_{(j_{u},j_{v}),x}}{\sqrt{l_{(j_{u},j_{v}),x}^{2}+l_{(j_{u},j_{v}),y}^{2}+l_{(j_{u},j_{v}),z}^{2}}}\right)$$

$$a_{(j_{u},j_{v}),y}=\arccos\left(\frac{l_{(j_{u},j_{v}),y}}{\sqrt{l_{(j_{u},j_{v}),x}^{2}+l_{(j_{u},j_{v}),y}^{2}+l_{(j_{u},j_{v}),z}^{2}}}\right)$$

$$a_{(j_{u},j_{v}),z}=\arccos\left(\frac{l_{(j_{u},j_{v}),z}}{\sqrt{l_{(j_{u},j_{v}),x}^{2}+l_{(j_{u},j_{v}),y}^{2}+l_{(j_{u},j_{v}),z}^{2}}}\right)$$

#### 3.1.1. Methods for Rearranging Joints

**Figure 3:**
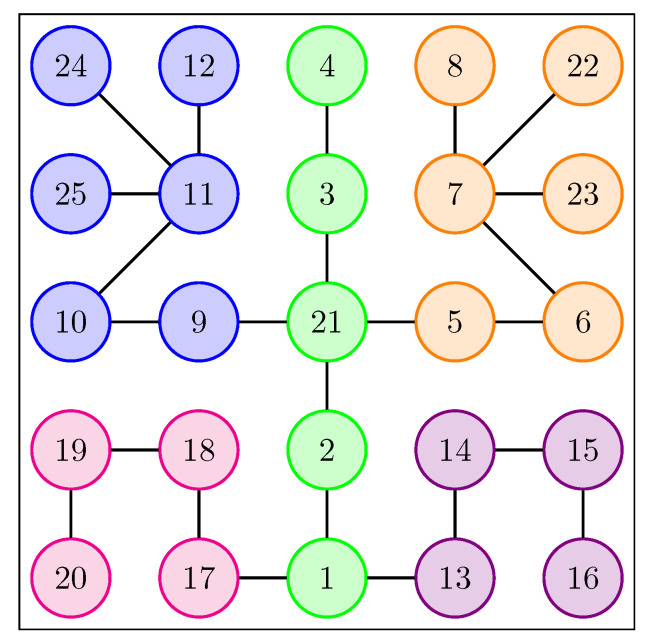
A proposal to rearrange the joints in a 2D format.

**Figure 4:**
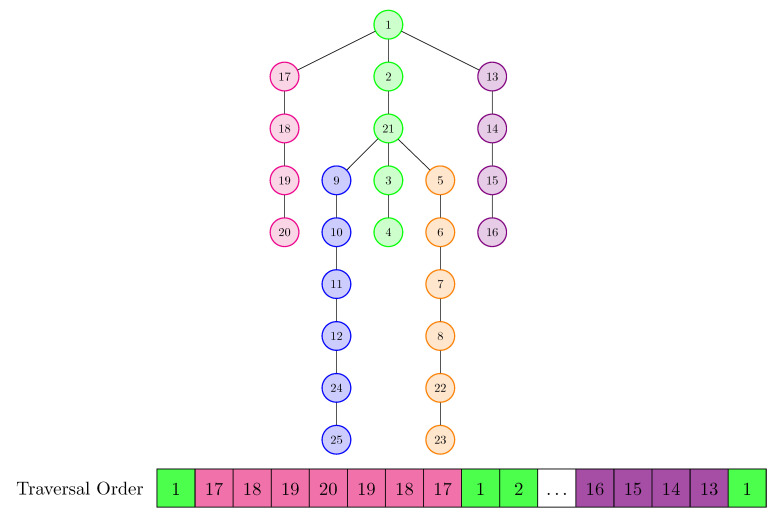
Transformation of the skeleton into a tree having the root of joint 1 (considered the centre of gravity). This tree is used for linearizing the skeleton (made based on a depth traversal applied to the tree).

To extract spatial dependencies, we proposed two variants of reorganizing the joints: one 2D (shown in Figure 3) and one 1D (shown in Figure 4).

The 2D variant was proposed earlier in our paper [11] and is based on a 5×5 matrix. The 2D variant presented in the Figure 3 allows the application of a TCN type layer based on 3D convolutions. This variant of representation considers the 5 essential parts of the body—left hand, torso, right hand, left foot and right foot.

The second proposed reorganization is a linear one and is inspired by Yang et al. [31]. We chose as the root for the proposed tree the central joint (the one with index 1), considering that it has a special importance, reason for which it was also used for the normalization step. The proposed tree is shown in Figure 4 and contains all 25 joints. We also considered the order in which the sub-trees for the root were added. Starting from this tree, we made a linear rearrangement of the joints starting from the DFS (Depth-first search) traversal of the tree. In this way, we made sure that any two nodes that appear side by side in the arrangement are also adjacent in the skeleton graph.

**Figure 5:**
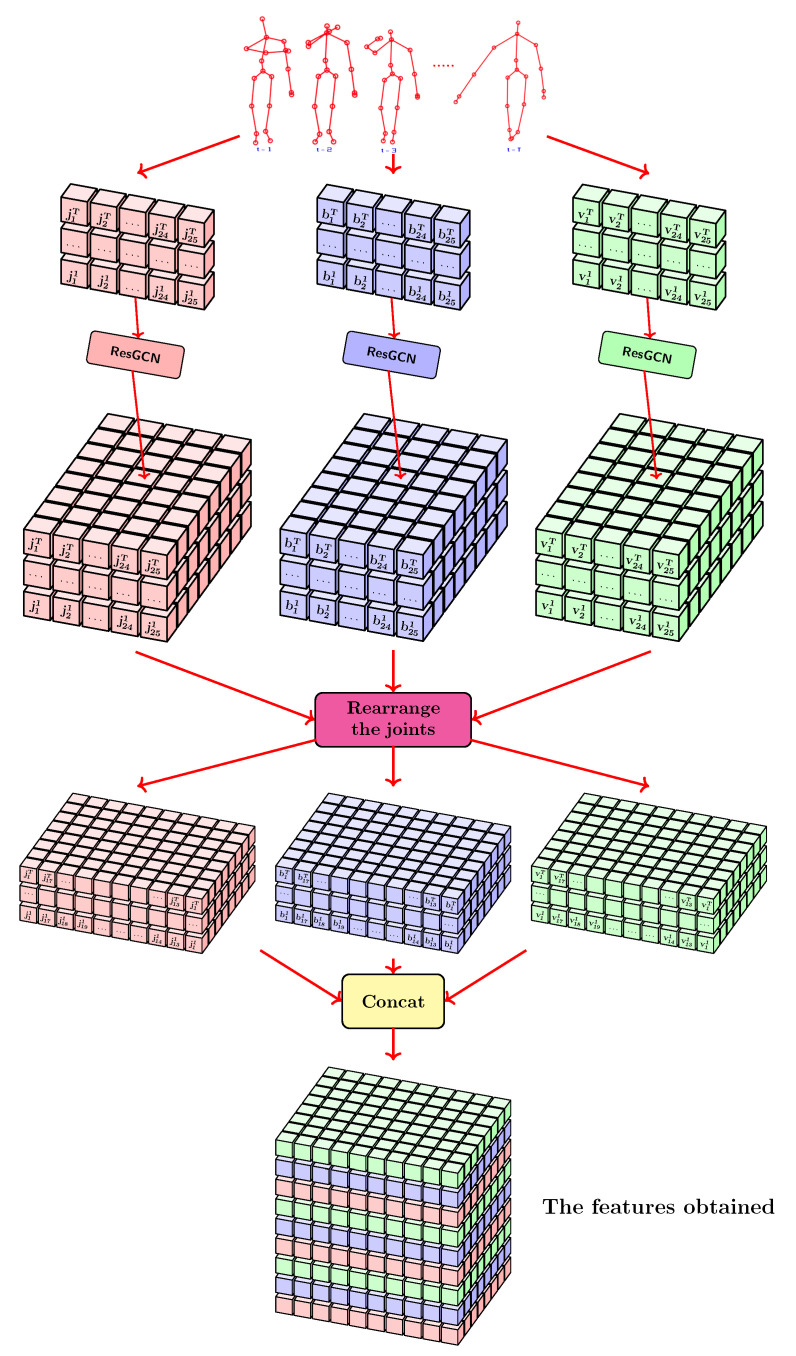
The pipeline that describes how to extract the features in the approach proposed by us.

In this paper, we use extensive variations of existing methods for rearranging joints. The main purpose of using this spatial rearrangement technique is to help the classification module work with relevant spatial features. In this proposed variant, the adjacent joints in the graph describing the skeleton appear adjacent in the feature vector. The novelty of our approach consists in the correlation of this method with TCN type layers. These layers apply dilated convolutions to the space domain. Figure 5 shows the entire flow that is applied to extract the features from the coordinates of the 25 joints. The features calculated using the formulas presented in Section 3.1 were extended by using Residual Graph Convolutional Network (ResGCN) layers. The described pipeline shows how the application of linear rearrangement influences the spatial arrangement of features. The features highlighted in red in the figure are those related to the joints, those presented in blue are those related to the bones, and those coloured in green are those related to the velocity. This feature extraction pipeline is inspired by the one proposed by Song et al. [7].

Our contribution consists in integrating the joint rearrangement module in this pipeline. This module will allow the first TCN type layers to extract dependencies starting from joints at distance 1, then the following ones analyse joints at distance 2 and so on, depending on the dilated factor used.

### 3.2. TCN-Based Architectures

**Figure 6:**
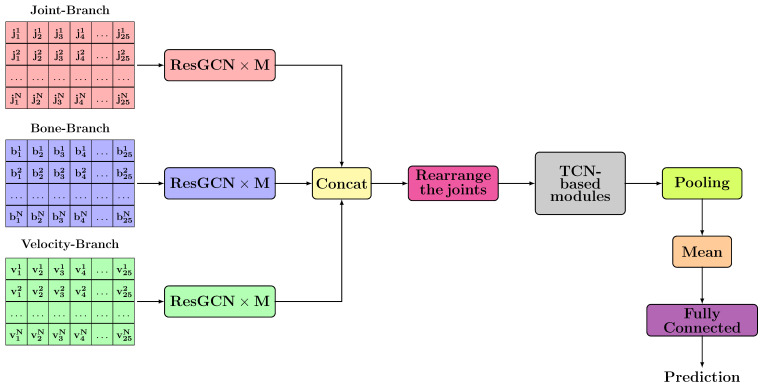
The proposed general architecture for Temporal Convolutional Network (TCN)-based approaches.

The general scheme of the proposed TCN-based architectures is presented in Figure 6. For each branch, *M* layers of ResGCN type are applied to extract spatial features. This part of extracting spatial features is inspired by the architectures proposed by Song et al. [7]. After spatial features have been extracted for each branch, we concatenate all features. Because we propose to use a module based on TCN type layers that will be able to extract both temporal and spatial features simultaneously, it is necessary to perform a rearrangement of the joints/bones. The proposed rearrangements are detailed in Section 3.1.1.

The TCN layers are modified so that they can be applied using 2D or 3D convolutions. This change is made in order to ensure the capture of spatial dependencies, in addition to temporal ones. Moreover, the dilated convolution is also applied to the axes related to the spatial dimension. Therefore, the order in which the elements related to the spatial dimension appear is important. After capturing these two types of dependencies that are synthesized by reducing their dimensionality based on dilated convolution, a pooling layer is used to achieve an average on the temporal axis and an average on the spatial axis.

As Figure 6 shows, there is a Mean block that signifies the reduction operation applied to the extracted features for the 2 skeletons. All actions were treated as some containing two skeletons, regardless of their type. It is important to note that we tried to consider the type of action (single-person action or two-person action), but the results were significantly weaker. This could be influenced by the errors that exist in the dataset, coming from the predictions of the Kinect sensor. In some samples in the NTU RGB+D dataset, the skeletons order changes from one frame to another. Another error of this type occurs when for single-person actions there are frames in which two skeletons are predicted. For example, the chair can be misinterpreted as a person and predicted as a skeleton. Thus, the operation applied to the features from each skeleton must be a commutative one.

#### 3.2.1. TCN-Based Modules

**Figure 7:**
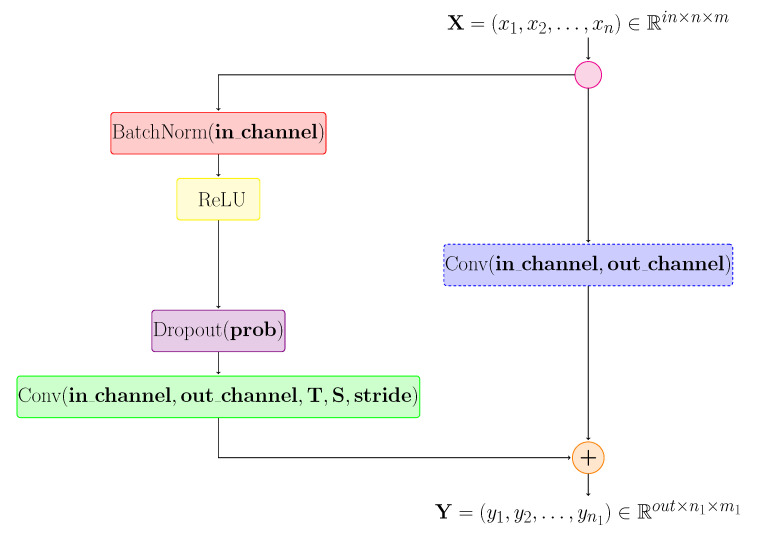
Architecture of a TCN type unit. The convolutional layer of the blue block is used only if in_channel≠out_channel. *T* represents the size of the temporal window, and *S* represents the size of the spatial window. If stride≠1 then $n_{1}<n$ and $m_{1}<m$, where *n* represents the number of frames and *m* represents the number of joints.

To analyse the sequence and extract features from a temporal perspective, we decided to use TCN layers. Thus, we started by testing several types of blocks based on TCN layers. Initially, we used a module based on TCN blocks inspired by the models previously proposed in [11]. Their major disadvantage was that they did not preserve the spatial size, because the TCN unit was based on 1D convolution. Therefore, we performed the concatenation of the extracted features for each joint. Then, we used the resulting 2D tensor as input for the TCN units. Figure 7 shows the architecture of such a TCN unit.

If the matrix representation method, previously presented in Section 3.1.1, is chosen then the convolutional layers used in the TCN unit will be 3D. For the additional convolutional layer, highlighted in Figure 7 by a blue block, we used value 1 for the kernel size.

**Figure 8:**
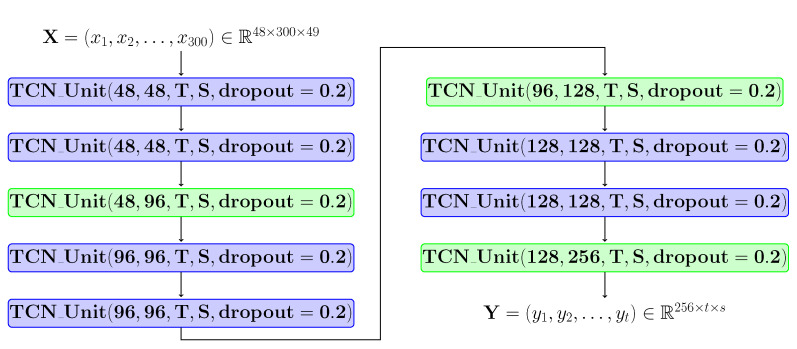
*T* represents the size of the temporal window, and *S* represents the size of the spatial window. For the blue blocks, the stride has the value 1, and for the green ones, the stride has the value 2. 300 represents the maximum number of frames, and 49 represents the number of analysed joints (results after the linear rearrangement described in Section 3.1.1). For each TCN type unit, the padding is determined based on the *T* and *S* values.

The blocks named TCN-based modules are highlighted in grey in Figure 6 and were made by composing several TCN unit layers. Figure 8 shows the structure of this module. TCN units for which no stride was applied are coloured in blue, and those for which stride was applied are coloured in green. For each block, the following information is specified: the number of input channels, the number of output channels, the kernel dimensions (temporal window and spatial window) and the probability with which the dropout was applied. Each block in this architecture follows the structure shown in Figure 7. After applying these TCN type units, a reduction operation will be applied to the temporal and spatial dimensions.

### 3.3. RNN-Based Architectures

**Figure 9:**
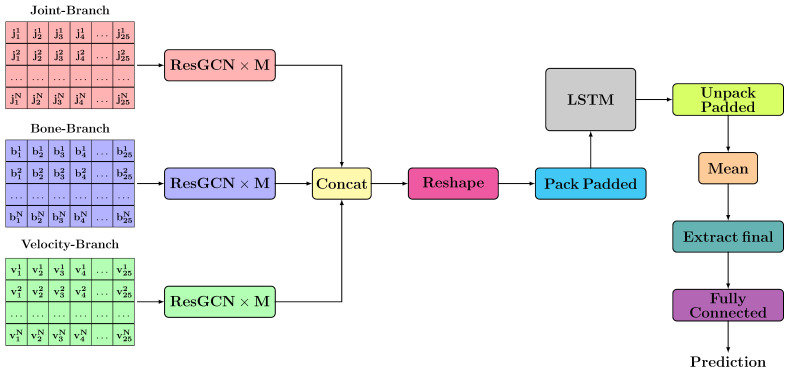
The proposed architecture for RNN-based approach.

The architecture used for the RNN-based approach is shown in Figure 9. This architecture is similar to the one previously presented in Section 3.2.1. To extract temporal dependencies, in this approach we used a module based on a multi-layer long short-term memory. Since we cannot keep the spatial dimension when working with an LSTM layer, we needed to apply a reshape operation on the tensor with features. Thus, the features used for each frame resulted from the concatenation of the features from each joint / bone. Because training and testing were performed using batches in which there were samples with a different number of frames, we used the two optimization operations that allow data rearrangement, considering the number of frames from each sample. These two optimization operations are represented in the Figure 9 by blocks Pack Padded and Unpack Padded.

In this architecture, the initial layers were applied independently for each skeleton. In the end, a mean of the extracted features was computed. Finally, only the features corresponding to the final hidden state for each sample are kept, and they are passed through a Fully Connected layer to achieve classification.

## 4. Experimental Results

### 4.1. NTU RGB+D Benchmark Dataset

NTU RGB+D [2,6] is a dataset that contains two versions—the first version contains 60 classes [6], and the second version added another 60 classes to those in the first version [2]. The actions in this dataset are actions that are performed by one or two people. All 120 types of actions are performed indoor, and 106 different subjects (aged between 10 and 57) contributed to the creation of this dataset. The actions included in this dataset can be divided into 3 main categories: daily actions, mutual actions, and health-related actions.

This dataset was recorded using 3 cameras positioned at the same height but placed at different horizontal angles: $-45^{\circ }$, $0^{\circ }$, $+45^{\circ }$. Each subject who participated performed each action twice: once towards the left camera, and once towards the right camera. Thus, a total of 114,480 samples were collected. Using a Microsoft Kinect v2, RGB images, depth maps, skeleton data (3D locations of 25 major body joints) and infrared images were collected for each sample.

For the first version of the dataset [6], the one containing 60 classes, two test protocols have been proposed—cross-subject and cross-view. The first protocol tests the ability of the model to generalize from the perspective of the person acting. The second protocol tests the ability of the model to generalize from the perspective of the camera position. For the second version of the dataset [2], the authors extended the cross-subject protocol but replaced the cross-view protocol with the cross-setup protocol. For the cross-subject protocol, some of the subjects were chosen for the training set, and the others for the test set. The first part of the dataset was collected using cameras that recorded from 3 perspectives. Two of them were used for training, and the third for testing. The entire dataset was recorded in 36 setups. For the cross-setup protocol, half of them were used for training and the rest for testing.

### 4.2. Implementation Details

A maximum of 50 epochs were used for each experiment. In our experiments, we tried to use an Adam or SGD type optimizer. We got the best results for the SGD optimizer with the Nesterov momentum and the following values for parameters: 0.1 for learning rate, 0.9 for momentum, 0.0002 for weight decay. For the learning rate, we tested several variants: different values without a scheduler (0.1,0.01,0.001 and 0.0001), decreasing the learning rate for each group of parameters based on a gamma value at each epoch or adapting the learning rate based on a Cosine type scheduler. The best performance was obtained for a Cosine scheduler for the learning rate with a warmup strategy [39]. For the Dropout type layers, we tested several values (0.1, 0.2, 0.3 and 0.5) and the best performance was obtained for a probability of 0.2. For the training, we used batches of 32 samples for all experiments. For all proposed models, the weights of the convolutional layers and the weights of the LSTM layers are initialized according to the Kaiming normal distribution [40], the batch normalization weights are initialized with 1s and bias with 0s. We performed all the experiments using two Tesla P100 PCIe GPUs.

### 4.3. Results

The results of the existing methods considered relevant together with the results obtained by the methods proposed by us are presented in Table 1. In this section, we explain the proposed and tested approaches using the NTU dataset, specifying for each one what are the particularities.

The values in Table 1 for the speed and the number of parameters presented for the models proposed by us are those determined for the Cross-Subject test protocol for the extended version of the NTU RGB+D dataset.

**Table 1:** Table showing comparative results from the perspective of accuracy for the four test protocols, but also performance related to computability (processing speed and number of parameters).

Method Name	Inference Speed ($\frac{\mathrm{sequences}}{\mathrm{seconds}\cdot \mathrm{GPU}}$)	Model Size (M)	Cross-Subject (NTU v1) Accuracy (%)	Cross-View (NTU v1) Accuracy (%)	Cross-Subject (NTU v2) Accuracy (%)	Cross-Setups (NTU v2) Accuracy (%)
HBRNN [41]	–	–	59.1	64.0	–	–
ST-LSTM [42]	–	–	69.2	77.7	55.0	57.9
TSRJI [43]	–	–	73.3	80.3	67.9	62.8
TSA [44]	–	–	76.5	84.7	67.7	66.9
VA-fusion [45]	–	24.6	89.4	95.0	–	–
ST-GCN [8]	42.9	3.1	81.5	88.3	70.7	73.2
SR-TSL [46]	14.0	19.07	84.8	92.4	–	–
PB-GCN [47]	–	–	87.5	93.2	–	–
RA-GCN [48]	18.7	6.21	85.9	93.5	74.6	75.3
GR-GCN [49]	–	–	87.5	94.3	–	–
AS-GCN [20]	–	6.99	86.8	94.2	77.9	78.5
2s-AGCN [19]	22.3	6.94	88.5	95.1	82.5	84.2
AGC-LSTM [34]	–	22.89	89.2	95.0	–	–
DGNN [50]	–	26.24	89.9	96.1	–	–
AS-GCN+DH-TCN [51]	–	–	85.3	92.8	78.3	79.8
SGN [52]	188.0	1.8	89.0	94.5	79.2	81.5
PL-GCN [53]	–	20.7	89.2	95.0	–	–
NAS-GCN [54]	–	6.57	89.4	95.7	–	–
ResGCN-N51 (Bottleneck) [7]	67.4	0.77	89.1	93.5	84.0	84.2
ResGCN-B19 (Basic) [7]	44.0	3.26	90.0	94.8	85.2	85.7
VPN [55]	–	–	–	–	86.3	87.8
DSTA-Net [56]	–	–	91.5	96.4	86.6	89
PA-ResGCN-N51 [7]	54.8	1.14	90.3	95.6	86.6	87.1
MS-G3D Net [57]	35.46	3.2	91.5	96.2	86.9	88.4
PA-ResGCN-B19 [7]	38.3	3.64	90.9	96.0	87.3	88.3
ResGCN-TCN (v1)—*ours*	94.29	3.14	89.06	93.81	84.1	84.58
ResGCN-TCN (v2)—*ours*	91.31	5.13	88.68	94.04	84.4	84.6
ResGCN-TCN (v3)—*ours*	97.03	2.13	88.05	93.29	84.0	84.15
ResGCN-LSTM—*ours*	121.85	2.15	85.01	92.3	79.93	81.28

The values for the speed and the number of parameters are taken from [7]; The inference speed is represented as the number of sequences processed per (second · GPU).

ResGCN-TCN (v1)—For this model, we used the linear rearrangement for the joints, the size of the temporal window was 9 and the size of the spatial window was 3 for all TCN blocks.

ResGCN-TCN (v2)—The difference between this model and the one presented above consists of the size of the spatial window. For this model, we used a spatial window of size 5 and this lead to a larger number of parameters. Due to the higher number of parameters, the speed decreased for this variant. From the perspective of the performances obtained for the test protocols, we did not discover notable differences.

ResGCN-TCN (v3)—This architectural model is similar to the previous one, the difference being the size of the temporal window. For this variant, a temporal window of size 3 was used for TCN units that do not use dilated convolution and a temporal window of size 9 for those TCN units that use dilated convolution. Thus, we obtained a smaller neural network, but with a performance comparable to the previous ones.

ResGCN-LSTM—As Table 1 shows, this model manages to get the best speed of all the proposed ones. The applied optimization is the one that allowed to obtain such a speed. From the perspective of the number of parameters, this is the model with the lowest number. However, because the spatial context is not preserved, this model obtains weaker results than the others for all analysed test protocols.

### 4.4. Ablation Study

Table 2 shows the impact that the joint rearrangement module has on the performance of the TCN-based model. We obtained the best results for the variant that uses the linear rearrangement of the joints. The model with the largest number of parameters is the one that uses the 2D rearrangement of the joints because for that model we transformed the 2D convolutions into 3D convolutions. For the first two variants, the reported results are obtained after a training process with 50 epochs, and for the last model, we increased the number of epochs to 70 due to the higher number of parameters.

### 4.5. Discussion

Among the previously existing methods, there is only one for which the number of parameters is lower and the inference speed is higher. This approach uses the Semantics-Guided Neural Network (SGN). This architecture contains two modules—a module that extracts features at the joint level and a module that extracts features at the frame level. The time required to train this approach was longer than the one used in the approach proposed by us—for the SGN-based version, 120 epochs were used, and for our proposed method only 50 epochs were used. The advantage of our approach is that it generalizes better. In other words, our method can discover features relevant to each action even when the number of classes is higher. This aspect allows us to obtain a better performance in the case of a dataset with a larger number of classes. Another advantage of our approach is that it does not consider the order of the skeletons. The Kinect sensor cannot keep the order of the predicted skeletons from one frame to another. In our architecture, the mean for the features obtained for each skeleton is applied at the end. This operation is commutative and allows the proposed approach to be invariant to the order of the skeletons. In the approach proposed by Zhang et al. [52], frames containing two skeletons are divided into two frames each containing a single skeleton. Because the Kinect sensor does not guarantee that skeletal order is maintained from one frame to another, this option may confuse the neural network.

The variants proposed by Song et al. [7] obtain better performances than our approaches for the NTU RGB+D dataset. An advantage that the solution proposed by Song et al. [7] has and that we will have to explore for the proposed approaches in the future is related to the explainability part. Even if the solution proposed by us achieves performance with a few per cent lower than PA-ResGCN-B19 [7], it has a higher inference speed which makes it suitable for use in a robotic framework that runs in a real-time scenario.

Another approach that achieves a better performance for the analysed dataset is the one proposed by Liu et al. [57]. The number of parameters for the MS-G3D model is comparable to those obtained in our approaches. It is relevant to notice that the inference speed obtained for this model is much lower. Unlike our approach, in the model proposed by Liu et al. [57], only the 3 coordinates of each joint are used as features. Even if the approach proposed by us uses a much larger number of features than the one used in the case of the MS-G3D architecture, it manages to obtain a lower inference speed due to the simple architectural model based on extended TCNs.

We tested a similar architectural model based on LSTM to highlight the importance of our methods based on an extended TCN type unit. Even if the inference rate is lower for the TCN-based approach, this aspect could be improved if the parallelization property of this type of neural network were used. Unlike RNNs, where the computations for later timestamps must wait for their predecessors to complete, convolutions can be computed in parallel even on simple and powerful SIMD architectures like those found in graphic cards because the same kernel or very similar kernels are independently used in each layer repeatedly. In contrast, in terms of performance, the best results were obtained for TCN-based approaches.

In the case of TCN-based methods, for samples that contained less than 300 frames, the padding operation is applied. In contrast, for LSTM-based approaches, this aspect is avoided by using specific optimization operations (e.g., Pack padded sequence in Pytorch). This may be one of the reasons why the inference speed obtained for TCN-based approaches is lower than that obtained when using LSTM.

An important advantage of TCN-based architectures is the ability to change their receptive field size in many ways. For instance, stacking more dilated (causal) convolutional layers, using larger dilation factors, or increasing the kernel size are all possible options, each with its specific advantages and disadvantages depending on the finer details of each implementation. This allowed us to use different values for the receptive field depending on the domain. The best performances were obtained when we used a kernel size equal to 5 for the spatial domain and a kernel size equal to 9 for the temporal domain.

## 5. Conclusions

In this paper, we presented two types of approaches that can solve the problem of recognizing human actions. These approaches are based on the two types of networks that are currently mostly used to determine temporal dependencies in sequence modelling problems—recurrent neural networks and temporal convolutional networks. In this paper, we first made a comparison between these two types of approaches from the perspective of the problem of recognizing human actions—a problem of modelling temporal sequences in which the spatial dimension is also very important. Based on the results obtained, we were able to highlight the fact that a model based on temporal convolutional networks is more suitable for this problem. One of the reasons why these approaches get better performance is related to the fact that they consider the spatial dependencies between the data. Thus, we have demonstrated that it is important to apply a rearrangement of the joints to allow the extraction of more relevant features in order to obtain a model with a greater ability to generalize. Being a problem that is often encountered in real-time running systems, we analysed the proposed methods from the perspective of the number of parameters and the inference time. The methods proposed by us have a smaller number of parameters and a better inference time than the methods with similar performances on the analysed benchmark. This fact makes them suitable for use in real-time scenarios. Moreover, the proposed TCN-based architectures are structurally simpler than others with comparable performance. This gives them a greater degree of generalization in terms of reuse for other types of problems. For this reason, they could be adapted and used for other types of time sequence modelling problems. Also, starting from the disadvantages of using the TCNs (e.g., the architecture may be dependent on the size of the sequence, difficult to work with sequences of different lengths), we demonstrated through the proposed methods how some of them can be diminished.

For future work, we have two directions of research. The first one relates to the proposal of an architecture that combines the properties of the two types of networks—LSTMs and TCNs. Such an approach could be useful for the situation in which one proceeds to analyse a sequence that describes an activity (e.g., a mixture of actions). It can be very difficult to detect when one action ends and another begins for a TCN-based approach. This would be necessary to achieve our final goal—to create a proactive social robot capable of understanding complex activities. The second direction is related to the extension of the recurrent neural network of LSTM type to take into account the spatial dimension. In this case, the format of the input received by the LSTM cell should be changed. For the problem of recognising human actions, the format used should be batch_size×time_steps×features×joints. Such an architectural modification would be useful for activities with a variable number of people.

Moreover, another further development consists of the integration of the proposed module within the Amiro robotic platform presented in [12]. In this way, we will be able to test the performance of the proposed methods in real-time running scenarios.

## Figures and Tables

**Table 2:** Results that show how the rearrangement of the joints influences the performance of the proposed architectural model.

Method Name	Cross Subject (NTU v2) Accuracy (%)
ResGCN-TCN—without rearrangement	83.49%
ResGCN-TCN—with linear rearrangement	84.4%
ResGCN-TCN—with 2D rearrangement	83.14%

## Abbreviations

The following abbreviations are used in this manuscript:

TCNs	Temporal Convolutional Networks
RNNs	Recurrent Neural Networks
GCN	Graph Convolutional Network
CNN	Convolutional neural network
GRU	Gated recurrent unit
LSTM	Long short-term memory
ST-GCN	Spatial-Temporal Graph Convolutional Networks
SGD	Stochastic gradient descent
ResGCN	Residual Graph Convolutional Network
